# Women alone modern contraceptive use decision making and its correlates, evidence from PMA CS 2021 survey

**DOI:** 10.1186/s12905-024-03050-x

**Published:** 2024-03-30

**Authors:** Solomon Abrha Damtew, Fitsum Tariku Fantaye

**Affiliations:** 1 Wolaita Sodo University, Wolaita Sodo, Ethiopia; 2 FTF research Consult., Addis Ababa, Ethiopia

**Keywords:** Contraceptive decision making, Married women, PMA Ethiopia

## Abstract

**Background:**

Women alone contraceptive decisions making has become one of the top burring public health agenda. Despite Contraceptive method options are available and accessible, contraceptive prevalence rate (CPR) in Ethiopia is not far beyond 41%. Evidences showed that the freedom of women to choose the contraceptive method they desired to use is one of the potential determinants for the sluggish pace of increase in contraceptive usage. In this era of sustainable development, determining the level of women own contraceptive use decision making and identifying its correlates is very critical for the ministries and relevant partners’ effort in tracking the achievement of Sustainable Development Goal (SDG) 5.2 by providing actionable evidence through informed decision-making with the aim of improving contraceptive uptake; reducing maternal mortality and improve newborn health.

**Methods:**

Nationally representative cross-sectional data from Performance Monitoring for Action (PMA) 2021 was used in this study. The sample was restricted among2446 married women who have been using or most recently used modern contraceptive method. Cell sample size adequacy was checked using a chi-square test. Frequency was computed to characterize the study participants. Multilevel binary logistics regression was used to identify factors associated with women own contraceptive use decision making. The findings were presented in a form of frequencies, percentage and as an odds ratio using 95% confidence interval. A *p*-value of 0.05 was used to declare significance.

**Results:**

This study revealed that higher than one in two women (59.49%; 95% CI: 57.7-61.38%) decide their contraceptive use by themselves. What is more interesting is that 1 in 16 women (6.06%) reported that they did not participated in their contraceptive use decision-making.-. Women aged 20 to 24 years; (AOR: 2.51 (1.04, 4.45)), women who stayed10 and above years in marriage; (AOR: 1.73 (1.08, 2.77)), whose husband and/or partner age is 41 and above years; (AOR: 2.14 (1.06, 4.31)) and those who obtained contraceptive method they desired; (AOR: 2.49 (1.36, 4.57)) had higher odds of deciding their current and/or recent contraceptive use by their own. On the other hand, women mixed feeling if they became pregnant at the time of the survey; (AOR: 0.6 (0.44, 0.91)), women who started using contraceptive at younger age, 19 to 24; (AOR: 0.6 (0.44, 0.81)), those who use long acting and/or permanent method; (AOR: 0.54 (0.41, 0.71)) and those married at younger age, 10 to 19 years; (AOR: 0.28 (0.09, 0.86)) had lower odds of independently deciding their current and/or most recent contraceptive use.

**Conclusion:**

59% of women independently decide their contraceptive use which calls up on further improvement to enable each woman to decide by their own, with directing special focus for the 6.06% of women who reported no say in their contraceptive use decision. Activities targeting on enabling women to use the method they preferred, spacing their pregnancy, encouraging women to discuss with their husband on the time and type of contraceptive method they used, advocating and promoting marriage at least to be at the minimum age as indicate by the law and maintain the marriage duration as much as longer are hoped to improve women alone contraceptive use decision making to the fullest.

## Background

Modern contraceptive use decision making refers to the ability of a women freely choose the contraceptive method they desired to use through the process of informed decision-making by effectively minimizing unnecessary pressure from important others around them [1–5]. Contraceptive method decision-making has received insufficient attention until recently since the priority of health policies, programs and researchers focused on availability, accessibility and utilization of contraceptive method by availing commodities near to the community at lower level through health posts. Similarly research and researchers focused on the factors that are influencing women contraceptive utilization. Such policies, program and research inclination were speculated by the Primary Health Declaration, Save the Mother initiatives in low- and middle-income countries [6–9] as indicators of the MDG era and the focus of Health Sector Transformation plan (HSTP) II [7]. However, in this era of SDG, however, women empowerment on contraceptive method and other reproductive health services uptake have attracted attention on a worldwide scale in terms of policy articulation, program design, and implementation, as well as monitoring efforts that have been influenced by researchers [7, 10–25].

Although massive efforts were made, most Sub-Saharan African countries, including Ethiopia, were not able to altered or improve their fertility rates overall or increase their optimum use of services, particularly contraceptive method which is attributed the slower prevalence rate which in turn is due to minimal women alone decision making on contraceptive use [26–28]. Recent evidences showed that women alone decision-making towards contraceptive use in particular; and reproductive and maternal health services in general is one of the key factors influencing service use, thereby contributing its share for the lower rate of contraceptive prevalence and high fertility rate [28–36]. As a result, though reproductive, maternal, neonatal, and child healthcare care in general and contraceptive method in particular are available, easily accessible, and mostly free of charge in Ethiopia as well [6, 25, 28, 37–40], uptake is not always ideal.

Therefore, during the last seven years, the global and the national communities have shown a paradigm shift in ensuring the optimum use of contraceptive services [41]. This is shown by the fact that goal 5.6.1 of the women’s empowerment main agenda lists the right of women to make decisions about contraceptive as a top SDG sub-agenda [8, 9, 24, 42] In Ethiopia, contraceptive method decision making received minimal or no attention likewise. By promoting reproductive health services use empowerment, including contraceptive use decision making, the Ethiopian government is committed in achieving the Sustainable Development Goal (SDG) to improve maternal health, lowering the maternal mortality ratio (MMR) from 401 to 279 per 100,000, and increasing the contraceptive prevalence rate (CPR) from 41 to 50% by 2025 [7]. Most reproductive health services are now available and easily accessible to the population due to several programs and initiatives launched and carried out by the Ethiopian Ministry of Health. For instance, provision contraceptive commodities, most maternal newborn and child services free of charge [6, 7]. The government further show it commitment by giving focus for empowerment in the recent HSTP II [7] and designing a separate SRH strategy running from 2021 to 25 [8].This suggests that in addition to making services accessible and available, it is necessary to examine the level and factors influencing women alone contractive use decision making, which are bottlenecks that impact service utilization [43]. Nevertheless, the factors were not clearly investigated in the previous research, therefore it is crucial to address any other possible factors, such as group related variables among others. Accordingly, generating and availing actionable evidence on contractive use decision making for the government and relevant partners, by determining the proportion of women alone contraceptive use decision making, and identifying its correlates would contributes for the achievement of the SDG indicator 5.6.1. This in turn contributes for the increasing national CPR improvement thereby contributing for the decrement of maternal and newborn mortality, and enhancement of women’s empowerment regarding the use of sexual and reproductive health services in general and contraceptive use in particular.

## Methods

### Study design, and population

Cross-sectional data from Performance Monitoring for Action Ethiopia (PMA Ethiopia) which was collected in the month of Nov 2021 survey were used for this analysis. PMA Ethiop collects nationally representative cross sectional and longitudinal data on maternal, new born health along with Family planning, women empowerment/norms and other relevant reproductive health indicators. It was carried out by Addis Ababa University’s School of Public Health in collaboration with the Ethiopian Public Health Association and Ethiopian Statistical services with financial support from Melinda Gates Institute for Population and technical support from Reproductive Health (Johns Hopkins Bloomberg School of Public Health), JHSPH.

Women in reproductive age groups who are married and/or cohabitated and who responded to the female questionnaire constitute the study’s source population. The analysis is restricted to women who are currently using and/or recently (within the last two years) used modern contraceptive methods. A total of 2446 (weighted count) women were included in this analysis.

### Sample size and selection techniques

The overall weighted count for this analysis 2446 was found adequate to generate reasonably unbiased estimate. Cell sample size adequacy was checked using chi square test.

The PMA-Ethiopia cross sectional and longitudinal survey was conducted among all women aged 15 and 49 residing in the selected households between. PMA Ethiopia is using a two-stage stratified sampling. A complete census was carried out in the chosen enumeration areas, after which 35 households per enumeration area were chosen using random number generator. After the household survey, all women of reproductive age were questioned.

Under the framework for the 2019 Ethiopia Population and Housing Census (PHC), which was conducted by the Ethiopia Central Statistics Agency, the primary sample units, or enumeration areas (EAs) were selected. As observations are picked using a method other than simple random sampling, the sample data is neither uniform nor randomly distributed. This method, commonly referred to as complex survey sampling, involves a range of selection probabilities at several stages. Each person’s weight is inversely correlated with the chance of selection. Estimate makes use of sampling weights that are included with the survey data rather than a straightforward random sample weight. Using independent selection in each sample stratum and a probability proportional to EA size, a total of 243 EAs were selected in the first stage. In the second round of selection, 35 HHs per cluster were randomly selected using a random number generator program from the newly created household listing. The interview was open to all females between the ages of 15 and 49 who were either permanent member of the selected HH or visitors who slept there the night before the survey. All of the information on sample design and selection techniques is included in the protocol of PMA Ethiopia [44].

### Study variables

#### Dependent variable

“Women alone contraceptive decision making” was the study’s outcome variable. The dependent variable question ‘who made the final decision about what method you got?’, with five response categories was dichotomized for analysis purposes into “important others = 0” (for married/cohabitated reproductive age women who reported that the decision on their FP use was made mainly by provider, partner, you and provider, you and partner and other) and “you alone = 1” (for married/cohabitated reproductive age women who reported that the decision on their contraceptive use was made only by themselves), (Table 1 below) [45].

**Table 1:** Description of the dependent variable

FP Decision Making	**Variable**	**Question & Responses**		**Categories**
		**Item**	**Response**	
	FPDM	who made final decision current/recent method	You alone = 1	1 = You alone
			Provider = 2	0 = Important others
			Partner = 3	
			You and Provider = 4	
			You and Partner = 5	
			Other = 96	

#### Independent variable

Potential confounders: Individual women characteristics, husband sociodemographic characteristics, contraceptive method related characteristics and group or enumeration area related variables (both integral and derived) were considered in this analysis.

Composite variables were created for contraceptive knowledge, contraceptive mass to mass media exposure, experiencing IPV, husband forced pregnancy.

“Contraceptive knowledge” was generated by sum up responses to the nine contraceptive knowledge questions and further categorizes into three groups; 1= ‘poor knowledge’ if respondent heard of 1–3 contraceptive methods, ‘moderate knowledge’= 2 if they knew 4–6 contraceptive methods and 3= ‘good knowledge’ if respondents heard of 7 to 9 contraceptives.

“Contraceptive exposure to -mass media” was formed from the variables (watching tv, listening to radio, and reading the newspaper and social media about contraceptives methods). As a result, women who watch TV, listen to the radio, or read on social media or newspaper about FP at least once were classified as having exposure to mass media (coded = Yes “1”), whereas those who did not do any of those things were classified as not having exposure to the media (coded = No “0”) [45].

“Husband/Partner forced to became pregnant” variable was created by sum up three variables and categorized 0= ‘not force’ if none and 1= ‘forced pregnancy’ if whether the respondents reported that her husband/partner forced her by treated by will have a baby with other women, will leave her, and forced her to get pregnant [45].

#### Analysis

Two data sets, namely, household, and female respondent were used for this study. STATA v16 was used for this analysis. Frequencies and percentages were computed to characterize the study population. Chi-square test statistics was computed to see the overall association/relationship of the independent variables with the two categories of women alone decision making. And it is also used to check cell sample size adequacy.

Frequency was run for every variable to check item nonresponse rate and don’t know response which were later excluded from the analysis. Following these variables were recoded to create biological plausible categories. This is followed by checking distribution of the variable using mean and proportion whenever appropriate categories were merged to make cell sample size adequacy.

Multicollinearity was checked and no strong multicollinearity was detected except between EA level and HH level wealth index variables, parity and marriage duration variables, partner discussion before contraceptive use and partner knows contraceptive use. The correlation coefficient for these variables was 0.8611, 0.7297, and 0.7023 hence EA level wealth, parity, discussion before use and partner know excluded from the final model.

Multilevel binary logistics regression was used to identify important factors of women alone contraceptive use decision making. At bivariate analysis a *p* value cut of 0.25 was used to select candidate variable for multilevel multivariable logistics regression analysis [46]. Results were presented in the form of percentage, and odds ratio with 95% CI. Significance was declared at a significance level of 0.05.

Four models were run; the first was the intercept only model in which no factors is included following which intra cluster correlation coefficient (ICC) is calculated to check the level of clustering observation among enumeration areas (EAs). The clustering was found to be 32.2% which is far beyond the conventional cut of point, 0.5 for the fulfillment of independent observation assumption, hence, supports the use of multilevel logistics regression. In the second model individual level variables were included while in the third model only enumeration area level variables were included. In the final model both individual and enumeration area level independent variables were included. For each model ICC, Akaike and Bayesian information criteria (AIC and BIC) along with log likelihood was calculated to check for model fitness. Based on the result, the final model with lower AIC and BIC along with higher likelihood was selected as best fitted model from which the adjusted odds ratio was computed and reported. Percentage change variation (PCV) was calculated except for the null model as the intercept only model was used as a reference.

#### Data quality management and control

In PMA Ethiopia survey, data were collected by well experienced PMA filed staff, resident enumerators using smart phones by customized Open Data Kit (ODK) system called PMA collect which facilitates real time data generation and timely feedback. Standard piloted questionnaires prepared in three local languages (Amharic, Afan Oromo, and Tigrigna). Weekly error progress report and response, close follow up during listing, householder, and female questioner data collection along with 10% re_interview and random spot checkups were conducted by field supervisors.

PMA Ethiopia data have been cleaned for public use to ensure its appropriateness for this analysis, data response completeness was checked, and item response rate and necessary measure was taken. Data cleaning and quality before conducting different analyses techniques was be employed in this study to exclude the missing values in each variable.

#### Ethical consideration

This study involved a secondary analysis of de_identified data from the PMA Ethiopia. The PMA Ethiopia survey was conducted strictly under the ethical rules and regulations of world health organization and IIRB of Ethiopian Health and Nutrition Research Institute (EHNRI). Informed consent was obtained from respondents during the data collection process of PMA Ethiopia on data collection on Oct 2021. PMA surrey has been also conducted after obtained ethical approval from Bloomberg School of Public Health at Johns Hopkins University in Baltimore, USA.

## Results

A total of 2446 married/cohabitating women aged 15 to 49 who are currently or recently used modern contraceptive, included in this analysis.

**Table 2:** Distribution of independent variables by women, partner related characteristics, PMA 2021 (weighted, *n* = 2,446)

Variables	Category	Freq.	Percent
	15–19 years	138	5.63
	20–24 years	533	21.79
Age	25–29 years	655	26.79
	30–34 years	449	18.35
	35–49 years	671	27.44
	17 to 30 years	863	35.3
Husband/Partner Age	31 to 40 years	967	39.53
	41 years and above	616	25.17
	No Education	741	30.29
Education	Primary	1078	44.07
	Secondary Plus	627	25.64
	No Education	624	25.71
Partner Education (n = 2427)	Primary	1004	41.38
	Secondary Plus	799	38.26
**Residence**	URBAN	778	31.81
	RURAL	1668	68.19
Regions	Other Regions	64	2.6
	Amhara	704	28.79
	Oromia	1070	43.74
	SNNPR	314	12.82
	Addis Ababa	155	6.35
	Sidama	139	5.7
Religion	Orthodox	1187	48.54
	Protestant	693	28.33
	Muslim	521	21.32
	Others	64	1.82
**wealth quintile**	Lowest quintile	404	16.51
	Lower quintile	504	20.61
	Middle quintile	458	18.72
	Higher quintile	455	18.62
	Highest quintile	625	25.54
Marriage type (n = 2444)	Monogamy	2310	94.53
	Polygamy	134	5.47
	No child	58	2.59
One child	574	25.5	
Parity (n = 2249)	Two children	517	23
	Three and Four Children	613	27.28
	Five and above children	486	21.63
Fertility Intention (n = 2189)	No More Another child	606	27.68
	Intended Have Another child	1583	72.32
Feeling if got pregnant	Happy	785	36.09
	Mixed Happy	330	15.16
(n = 2176)	Unhappy	1061	48.75
Age at first use (n = 2419)	10 to 18 years	793	32.79
	19 to 24 years	993	41.04
	25 to 45 years	633	26.18
Number of children at first use	No child	910	37.73
(n = 2411)	1 to 2 children	1011	41.96
	More than 3	490	20.31
Contraceptive Knowledge (N = 2444)	Poor Knowledge	203	8.29
	Moderate Knowledge	1403	57.42
	Good Knowledge	838	34.29
Contraceptive exposure to	No Information	1372	56.38
mass-to-mass media (n = 2434)	one source	618	25.40
	More than one source	443	18..22
Contraceptive obtained desired	No	168	6.87
**(n = 2245**)	Yes	2277	93.13
Informed contraceptive side effects	No	1647	65.94
( **n = 2445)**	Yes	498	34.06
Discussion before use	No	614	25.10
	Yes	1832	74.90
Partner knows contraceptive use	No	220	9.05
(**n = 2435)**	Yes	2215	90.95
Visited By Health Worker	No	2218	88.14
(,=2433)	Yes	215	11.86
Husband forced pregnancy	Not Forced	2202	90.15
(n = 2443)	Forced	241	9.85
HH Members	1 to 3 members	695	28.43
	4 to 5 members	1364	44.17
	6 to 16 members	387	27.40

The summary result reveals that 1 in 4 (26.79%) of women aged 25 to 29 and their husbands were aged above 41 years (25.17%), whereas 25.64% of all women had secondary education and 25.71% of their husband had not education. It was discovered that women living in rural regions outnumbered their urban counterparts (68.19%), and 44.17% of households include four to five members. Orthodox Christianity was the most common religion (48.54%), followed by Protestantism (28.33%), while 1 in 6 (16.51%) of women fall into the lowest quintile and 43.74% reside in Oromia region. One in five (21.63) had five or more children, while 2.59% had none. Nearly ¾ of women (72.32%) said they intended to have another child, though 1 in 20 women (5.47) reported that they are in polygamy marriage.

In addition, 37.73% of women are current/recent family planning user did not yet have children at the time they began taking contraceptives for the first time, and 41.04% of them began using while they were between the ages of 19 and 24. Almost half of the women (56.38%) claimed having no information of contraceptive in the previous 12 months, while 57.42% reported that they had moderate knowledge on contraceptive methods. Women who reported getting the contraceptive method they desired and being told about any potential side effects of this technique were reported to be 93.31% and 34.06%, respectively, whereas women who reported having contraceptive side effects were reported to be at 32.82%.

Nine in 10 (90.95%) women indicated that their husbands and/or partners are aware of the contraceptive method they are using and 90.15% reported that their husband/partner forced them to become pregnant while ¾ (74.9%) of them reported that they discussed with their husband/partner before to using it (Table 2).

**Table 3:** Distribution of women by method related characteristics variables, PMA (weighted, *n* = 2446)

Variables	Category	Freq.	Percent
Told another Method	No	1210	49.48
(*n* = 2445)	Yes	1235	50.52
Couples Age Difference	Less than 10 years	1876	76.71
	Equal or greater than 10 years	570	23.29
Method Type	Short acting	1623	66.37
	Long acting	823	33.63
Visited a facility	No	922	37.87
(*N* = 2434)	Yes	1512	62.13
Experiencing Side Effects	No	1643	67.18
	Yes	803	32.82
Marriage Duration	lessthan_10_years	1309	53.51
	Morthan_10_years	1137	46.49
Age of Marriage	10 to 19 years	1721	70.38
	20 to 29 years	682	27.87
	30 and above years	43	1.75

In the last 12 months, 2/3 (62.13%) of women reported that they visited a facility, while half of them (50.52%) mentioned they were informed about another method. One in three woman (33.63%) of the women used long-acting contraceptives. One in 4 (23.29%) reported an age difference of 10 or more years and 27.87% engaged to marriage in the age group 20 to 29 years while 46.49% reported that they stayed in marriage for more than 10 years (Table 3).

### Level of modern contraceptive use decision making, PMA 2021 CS survey

**Fig. 1:**
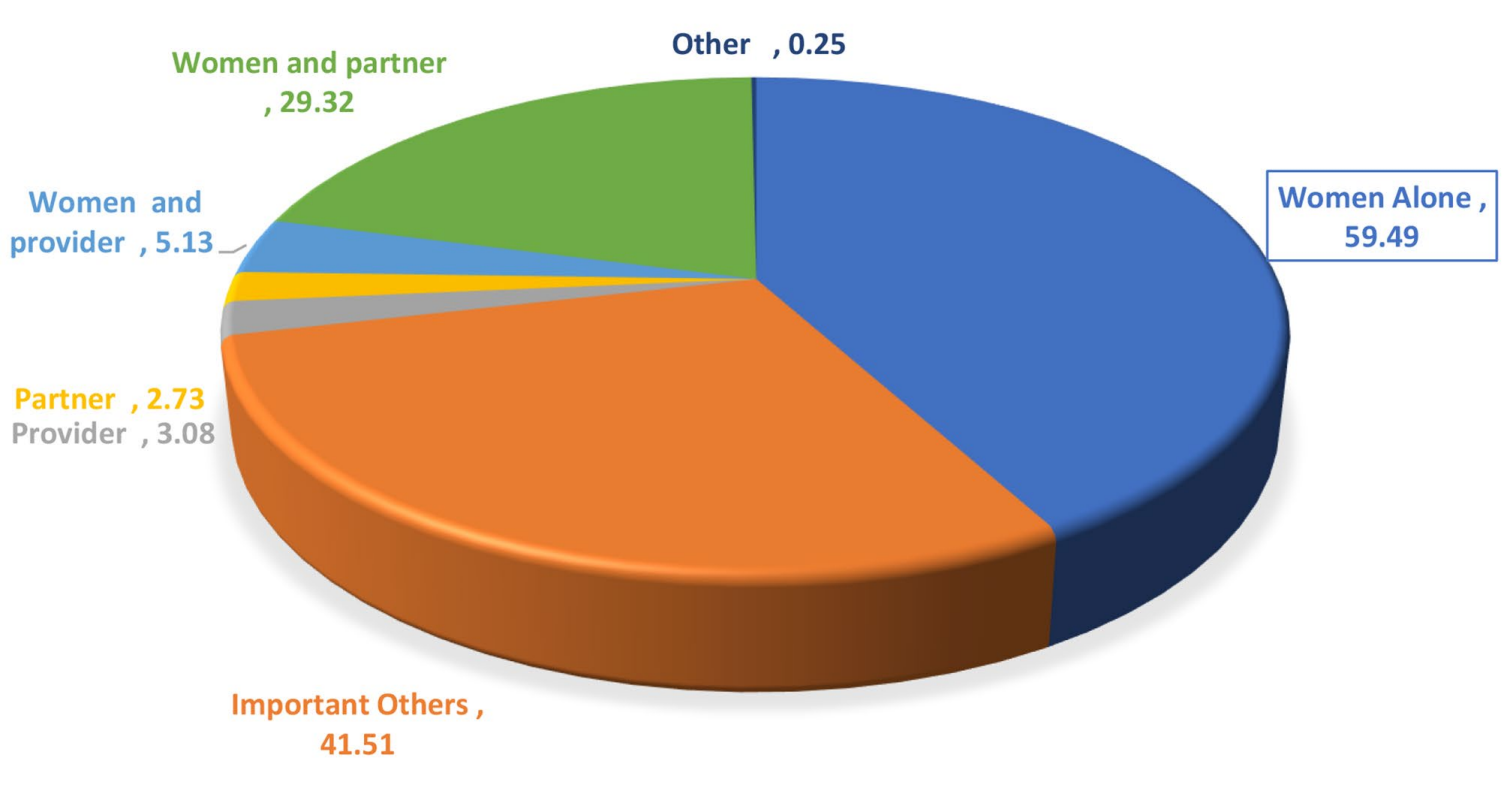
Level of decicion making among women, evidence from PMA 2021 CS Survey

Nearly 2/3 (59.49%; 95% CI 57.5–61.2%) of women decide their recent and current modern contraceptive use by themselves. Nearly 1/3 (29.32%) women decide their contraceptive use jointly with their husband and/or partner. One in 20 (5.13%) women decide with the provider while provider alone and husband/partner alone decision on woman contraceptive use was 3.08% and for 2.73% respectively. One in 16 women (6.06) reported that others decide their contraceptive use (Fig. 1 **above**).

**Table 4:** Multilevel Binary Logistics Regression Result for factors affecting Women alone contraceptive use decision making among women, Evidence from CS 2021 data

Variables	Categories	Null Model	Model II AOR 95% CI	Model III AOR 95% CI	Model IV AOR 95% CI
Age	15–24 years		1		1
	20–24 years		2.09 (1.02, 4.3)		2.15 (1.04, 4.45) *
	25–29 years		1.58 (0.76, 3.26)		1.62 (0.78, 3.36)
	30–34 years		1.38 (0.52, 3.67)		1.42 (0.53, 3.76)
	35–49 years		1.03 (0.38, 2.86)		1.05 (0.38, 2.92)
Education	No Education		1		1
	primary		1.09 (0.77, 1.54)		1.1 (0.78, 1.56)
	Secondary Plus		1.24 (0.75, 2.07)		1.25 (0.74, 2.12)
partner education	No Education		1		1
	primary		0.86 (0.60, 1.25)		0.9 (0.62, 1.32)
	Secondary Plus		0.66 (0.43,1.01)		0.7 (0.45, 1.10)
wealth quintile	Low		1		1
	Middle		0.76 (0.2 ~ 52, 1.12)		0.76 (0.52, 1.10)
	High		0.91 (0.58, 1.44)		0.92 (0.56, 1.53)
Religion	Orthodox		1		1
	Protestant		0.69 (0.47, 1.01)		0.81 (0.53, 1.24)
	Muslim		1.43 (0.87, 2.35)		1.44 (0.87, 2.39)
HH Members	Less than 2		1		1
	1 to 3 members		1.33 (0.47, 1.74)		1.11 (0.71, 1.71)
	4 to 6 members		1.33 (0.95, 1.88)		1.31 (0.93, 1.85)
Marriage type	Monogamy		1		
	Polygamy		1.38 (0.77, 0.25)		1.44 (0.79, 2.64)
Partner Age	to 30 years		1		
	31–40 years		1.36 (0.85, 2.17)		1.35 (0.84, 2.15)
	41 and above years		2.13 (1.06, 4.26)		2.14 (1.06, 4.31)*
couples age	< 10 years		1		
difference	≥ 10 years		0.88 (0.61, 1.27)		0.87 (0.60, 1.26)
Obtained desired	No		1		
FP method	Yes		2.93 (1.35, 4.51)		2.49 (1.36, 4.57)**
Get preg feeling	Happy		1		
	Mixed		0.61 (0.41, 0.91)		0.6 (0.40, 0.90)*
	Unhappy		1.01 (0.42, 1.41)		1.02 (0.73,1.43)
Future Fertility	No More child		1		
Intention	Have Another child		0.75 (0.51, 1.11)		0.75 (0.50, 1.11)
Age at first use	10 to 18 years		1		
	19 to 24 years		0.59 (0.43, 0.80)		0.6 (0.44, 0.81)***
	25 to 45 years		0.68 (0.45, 1.02)		0.69 (0.46, 1.03)
Current/recent	Short acting		1		
Method used	Long acting		0.54 (0.41, 0.70)		0.54 (0.41, 0.71)***
Marriage duration	lessthan_10_years		1		
	Morthan_10_years		1.73 (1.08, 2.78)		1.73 (1.08, 2.77)*
age_of_mariage_2	10 to 19 years		1		
	10 to 19 years		0.27 (0.090.85)		0.28 (0.09, 0.86)*
	20 to 29 years		0.35 (0.12, 1.05)		0.35 (0.12, 1.04)
Regions	Other Regions			1	
	Amhara			1.59 (0.75, 3.38)	1.37 (0.57, 3.28)
	Oromia			1.11 (0.56,2.24)	1.14 (0.50, 2.58)
	SNNPR			0.51 (0.25, 1.05)	0.68 (0.29, 1.60)
	Addis Ababa			1.89 (0.83, 4.29)	2.18 (0.83, 5.68)
	Sidama			0.53 (0.2, 1.45)	0.54 (0.17,1.78)
	Partner EA education	1.00		1 (0.98, 1.01)	1 (0.98, 1.01)
	Women EA education	1.00		1 (0.98, 1.01)	1 (0.98, 1.02)
	ICC EA_ID	32.8	30.4	29.8	29.6
	PCV	Reference	7.317073171	9.146341463	9.756097561
	Loglikelihood	. -1550.201	-1260.05	. -1540.139	. -1255.616
	AIC	3104.402	2580.101	3098.278	2585.232
	BIC	3116.007	2749.706	3150.498	2794.411

*= *p* value < 0.05 ** = *p* value < 0.01 *** = *p* value < 0.001

### Factors affecting women alone contraceptive use decision making

The intra cluster correlation coefficient (ICC) for the null model was 33.0% which indicates the contribution of the variation among EAs in which women reside for women alone contraceptive use decision making while the individual characteristic difference accounted for the remaining variation. This contribution reduced to 29.6% after accounting both individual and EA level variables. Moreover, 10.0% of the variation in women alone contraceptive use is explained by both the individual and EA-level variables as indicate in the final model (model IV). The best fitted model is model IV which has lowest AIC and higher log likelihood (Table 4).

An increased woman age, obtained the desired method, staying longer in marriage and having elder husband were found positively and significantly associated with increased odds women alone contraceptive use decision making. On the contrary, women who are using or used long-acting method, married at early age and mixed feeling if getting pregnant were found to have decreased odds of women alone contraceptive use decision making.

In this study an increase woman age was found to increase the odd of women alone contraceptive decision making by (AOR: 2.15 (1.04, 4.45)) compared with women aged 15 to 19 years. The other variable which was found to increase the odds of women alone contraceptive use decision making is marriage duration. Accordingly, staying in marriage and/or cohabitation more than 10 years increases the odds of women alone decision making on contraceptive use by (AOR: 1.73 (1.08, 2.77)). Similarly, in this study woman who have obtained the method they desired were found to have (AOR: 2.49 (1.36, 4.57)) much higher odds of deciding their contraceptive use by themselves. Likewise, women whose husband aged is 41 years and above have (AOR: 2.14 (1.06, 4.31)) higher odds of deciding their contraceptive method compared with women with younger husband aged 17 to 30 years.

On the other hand, using long-acting method were found to lower women alone contraceptive decision making. Accordingly, women who used long-acting method have (AOR: 0.54 (0.41, 0.71)) lower odds of deciding contraceptive use by themselves compared with those used short acting. In addition, women who married at younger age (10 to 19 years) have 72% lower odds (AOR: 0.28 (0.09, 0.86)) of deciding contraceptive use by themselves compared with those who engaged to marriage at later age, above 30 years. Similarly commencing to use contraceptive at younger age (19 to 24) have only (AOR: 0.60 (0.44, 0.81)) lower odds of deciding their contraceptive use by themselves compared with those who commenced at very earlier (10 to 18).

Finally, women feeling if get pregnant was associated to reduce the odds of women alone contraceptive use decision making. Hence, mixed feeling if get pregnant is associated with lower odds of contraceptive use decision making (AOR: 0.6 (0.40, 0.90)) by themselves compared with unhappy/sort of unhappy (Table 4).

## Discussion

In the era of the SDG contraceptive use decision making is one of the indicators of sustainable development goal (SDG) agendas, hence, determining the magnitude of women alone contraceptive use decision making level and identifying the correlates affecting in greater depth has paramount importance in monitoring the achievement of such burning global agenda. Unfortunately, studies conducted in Ethiopia to measure contraceptive use decision making are limited in number, geographical scope and do not account the EA level factors. Therefore, this study used nationally representative updated data to determine the magnitude of women alone modern contraceptive use decision making and identify correlates affecting it.

Accordingly, nationally closer to 6 in 10 (59.49%) of women reported that they decide their current or recent contraceptive use by themselves while 29.32% decide jointly with their husband and/or partner. One in 16 women (6.06%) reported that they were not participated on their current and/or recent contraceptive use decision, while 1 in 12 women (8.21%) reported that their decision on their current and/or recent contraceptive use was made jointly with the health care provider or decided by the health care provider alone. A set of factors which both positively and negatively influence women alone decision on their current and/or recent contraceptive use were identified.

The level of women alone decision making was in line with other studies 53.8 [3], 57% [47] and 52% [4]. It’s found higher that similar study 22% [48], 21.6% [49] 14.2% [1], 1/3 [4]. This might be related with outcome variable measurement difference [1, 4] and time difference and variation in the categories of the outcome variable where this study includes two additional categories (decision by health care provider alone and deicide jointly with the health care provides) [48, 49]. The prevalence of this study is higher than studies [50–52] conducted in high fertility regions of Ethiopia using EDHS data (17.2% and 23%). It was found higher than a study based on data from PMA 2020 analyzed using multinomial logistics regression as statistical modeling [53] which reported (51.2%; 95% CI: 48.8-53.6%) for women alone decision making. This same study reported an increase on women alone decision making from 2014 to 2020 accordingly, proportion of women who decided by themselves increased 32.8% (95% CI: 29.4%, 36.4%) in 2014 to 51.2% 95% CI: 48.8%, 53.6%) in 2020 which implies that and increasing trend over the last 8 years, such an increase is attributed to the successful accopmlememt of MDG goals along with health sector transformation plan I [25] and the Ethiopia Government commitment to achieve women and girls empowerment SDG goal as we are mid-way the SDG period [42].

The fact that only 6 in 10 women decide their contraceptive use by themselves imply that the world has a long way to go in fulfilling’s the complete implementation the human right approach in contraceptive provision [54]. Similarly, women contraceptive decision made by health care providers alone and with women will have an implication for the existing low contraceptive quality counseling with decreasing trend in good quality counseling [55, 56] which in in turn contributes for the staggering contraceptive prevalence rate. Move over, making women empowered in general and in contraceptive use decision making in particular as integral part of the national reproductive health strategy while revising the existing RH policy 2021 to 25 is another implication of the finding in this study [8]. Furthermore, the finding is critical to maintain the success of the London Family Planning Submit and contributes its share for universal access of contraceptive methods for women and girls [57].

Regarding the correlates of women alone contractive use decision making: Younger age, staying longer in marriage, obtaining desired method, living with elder husband and were found to increase the odds of women own contraceptive use decision making.

In line with studies [49–51, 58] increasing women age was found to increase the odds of women alone contraceptive use decision making while a study [2] reported that women aged 35 to 44 have lowers the odds contraceptive use decision making, yet another study [4] reported that women in the age group 18 to 20 were found with an increased odds of decision making. The discrepancy might be related with the scope of the study; this study used nationally representative data while the cited studies were conducted in a single woreda and outcome variable measurement approach, in this study contraceptive decision making was measured using a single question while composite variable was used in the studies cited. Yet another study [59] in Sub-Saharan Africa reported that younger women aged below 34 were found to have a lower odds of women alone decision making. The discrepancy might be attributed to the difference in the study design, sociocultural and economic difference between Ethiopia and other Sub-Saharan African countries included in the cited study.

Another factor that was found significantly increased the odds of women alone contraceptive use decision making was getting the desired method, accordingly, in line with a study [60] obtaining the method they desired was positively and statistically increase women alone contraceptive use decision making.

The two more correlates of women own decision making were staying longer than 10 years in marriage and having a husband older than 41 years and above. Living with a husband/partner aged 41 years and above was found to increases the odds of women alone decision making while a study [51] reported that having elder husband has not effect. In line with a study [58] staying long in marriage for 10 or more years was found to increases the odds of women own decision making. Similarly our finding is in line with a study conducted in south west Ethiopia, Mettu District [13].

Starting to use contraceptive at younger age, using long and/or permanent method, engaged to marriage at younger age and mixed happy and unhappy feeling if they became pregnant by the time of the survey were found to lower the odds of women alone decision making. In line with [49] engaging to marriage in the early age was associated to lower the odds of women alone decision making. Obtaining long acting and/or permanent methods found to lower the odds of women alone contraceptive use decision making. In agreement with studies [61, 62] and in line with findings form [34, 63] respectively using long acting and mixed feeling if they got pregnant by the time the survey were found to lower the odds of women alone contraceptive use decision making. This might be related with the prestige of having more children is social acceptable and the cost of care for more children [30].

In contrary to studies, educational status, residence [2, 4, 32, 47, 49, 51, 58], wealth quintile, EA level variables such as EA wealth, proportion of women and husband who completed secondary education and above, future fertility desire, women knowledge on contraceptive methods [1, 3, 4, 13, 49, 58], women place residence [5] were not found to be associated with women alone contraceptive use decision making. This might be due to the difference in sample design, scope of the study and how the outcome variable was measured (some generated and used composite variables and some use single variable). The other possible likely reason is the inclusion of more confounders in this study unlike those cited here in and one study focus on knowledge, attitude and overall awareness related factors that affect women contraceptive use decision making [3]. Similarly, unlike studies [4, 49, 52] exposure to media is not significant in this study.

This study is not spared from limitations. To begin with, data on potential confounder variables such as husband desired number of children and timing of additional child, and women and husband employment were not collected by PMA survey. In addition, as a matter of fact, PMA Ethiopia 2021 cross-sectional survey did not collected data from Tigray region due to the conflict, therefor any form generalization need to consider this in mind. As a strength this study address potential confounder variables, notably group level variables.

## Conclusion

To start with, nearly 6 in 10 women who are using currently and/or used recently modern contraceptive method were able to decide their contraceptive independently which calls further improvement to escalate up and maintain near to 100%. To our surprise 1 in 12 women (8.21%) women decide their contraceptive use with the health care provider: provider alone and jointly with the provider accounted for 3.08% and 5.13% restively. What is more interesting is that 1 in 16 women (6.06%) reported that they have no any say in decide ding their contraceptive use. In addition, Polices, strategies and interventions that are designed to enable every women who intended to use contraceptive method to independently decide and the contraceptive method she wanted to use are hoped to help women fully decide by themselves without anyone´s interference and reduces the effect of others. In addition, efforts need to be made that mitigate any form of negative influence from husbands and/or partners and significant others will create conducive environment for the women to decide by themselves. Women alone decision making on contraceptive is likely to be improved by efforts that help women to use contraceptive method based on detail information on methods available during their contraceptive use visits. Providing advice when to get pregnant (planning and spacing pregnancy) also contribute their share in improving women alone decision making.

Furthermore, the finding that younger women had higher odds of decision making and those who married at younger age had lower odds to decide by themselves which calls up on creating awareness on reproductive health service availability and where to utilize it including maximizing the use of youth friendly service access and counseling on the minimum age of marriage as per the Ethiopian family low thereby empowering young/adolescent girls to use contraceptive method. Activities targeting on enabling women to use the method they preferred, spacing their pregnancy, encouraging women to discuss with their husband on the type of contraceptive method they used; that advocating and promoting marriage at least to be in the minimum age as indicate by the law and maintain the marriage duration as much as longer are hoped to improve women alone contraceptive use decision making to the fullest. Finally, including missing variable such as husband desired number of children and timing of additional child, husband employment, women employment for future research in general and performance monitoring for action (PMA) survey in particular.

## Data Availability

The datasets used and/or analysed during the current study are available from the corresponding author on reasonable request.
